# Association mapping for hop cone chemistry and morphology identifies natural beneficial allele stacks

**DOI:** 10.1002/tpg2.70238

**Published:** 2026-04-28

**Authors:** Shaun J. Clare, Peter Schmuker, Kayla Altendorf

**Affiliations:** ^1^ Department of Crop and Soil Science Washington State University Pullman Washington USA; ^2^ USDA‐ARS Forage Seed and Cereal Research Unit Prosser Washington USA

## Abstract

Efforts are underway to increase the efficiency and precision of selection hop (*Humulus lupulus* L.) breeding using genomics. Little is known, however, about the genetic control of important traits like α‐and β‐acids contents, oil content, and cone morphological characteristics, all of which play an important role in determining the utility and harvestability of a hop and are targets of selection. In this study, we utilized association mapping with a collection of 529 female hop plants evaluated in Prosser, WA USA in 2023 and 2024, single nucleotide polymorphism data derived from genotyping‐by‐sequencing with 20,861 markers, and phenotype data generated from near‐infrared (NIR) spectroscopy and image analyses of hop cones. A total of 49 significant marker trait associations were detected across five traits with 43 unique loci. High correlation estimates between wet lab and near‐infrared spectroscopy data (*R* = 0.54–0.94), high broad‐sense heritability estimates (*H* ^2^ = 0.32–0.71), and logical associated candidate genes illustrate the validity of the methods used in detecting meaningful associations. Furthermore, existing germplasm in our study containing increasing stacks of favorable alleles showed improvement in all traits, demonstrating the potential for utilizing the markers identified herein in a genomic prediction pipeline to improve hop germplasm for key end‐use traits.

## Core Ideas

- NIR is a rapid and cost‐effective method to measure cone chemistry traits in large populations of hop.
- Association mapping identified key loci involved in cone chemistry and morphology traits.
- The majority of significant markers exhibited a clear additive phenotype.
- Natural beneficial allele stacks are present within the breeding program that can be further exploited.
- Hop exhibits potential for the application of genomic selection.

## Plain Language Summary

Hop is a key ingredient in beer as it provides bitterness, aromatics, and flavor. The regions of the hop genome that control these important attributes, such as α‐acids, β‐acids, total oil content, as well as the shape and size of the cones from which these compounds are derived, have not been thoroughly investigated, thereby limiting the use of highly efficient genomics‐assisted breeding methods. This study identifies regions within the hop genome that are associated with increases in α‐acids, β‐acids, total oil content, and improved cone shape and size for increased harvestability. These findings demonstrate the use of higher‐throughput methods for measuring cone chemistry using near‐infrared (NIR) spectroscopy and cone morphology using image analysis. The results provide insight into the genetic control of important traits and demonstrate the potential for the use of genomics‐assisted breeding in hop to increase the efficiency of cultivar development.

**Abbreviations**
AM: association mapping
BLINK: Bayesian‐information and Linkage‐disequilibrium Iteratively Nested Keyway
BLUE: best linear unbiased estimate
BLUP: best linear unbiased prediction
GAB: genomics‐assisted breeding
NIR: near‐infrared spectroscopy

## 1 INTRODUCTION

Knowledge of the genetic control of plant traits is a critical step for implementing genomics‐assisted breeding (GAB) to improve the efficiency and precision of selection (Varshney et al., 2005). Long‐lived perennial species with extended juvenility and prolonged cycle times, complex genomes, and costly infrastructure requirements can sometimes be intractable for implementing GAB. Such species also have considerable potential gains in terms of breeding efficiency, a challenge sometimes referred to as the “perennial problem” (McClure et al., 2014). Breeding of hop (*Humulus lupulus* L.), a perennial species used primarily in the brewing of beer, with a historically long (10–15 years) cultivar development timeline, faces many of these challenges (Haunold, 1981).

Hop is an outcrossing species with a complex dioecious mating system (2*n* = 2*x* = 18 + XX/XY) (Padgitt‐Cobb et al., 2023). The strobiles of the female plant (or cones, to which they are commonly referred) contain glandular trichomes, or lupulin glands, which are rich in flavor‐active secondary metabolites (Patzak et al., 2015). The vast majority of hop is produced and used for flavoring and bittering of beer in brewing (98%), with a small portion dedicated to pharmacological and medicinal uses (Korpelainen & Pietiläinen, 2021). Common hop (*H. lupulus)* is the only domesticated *Humulus* species and is the species utilized herein. Hop has been traditionally grown between the 45° and 52° parallels with most hops produced in Germany, the United States, the Czech Republic, Poland, and Slovenia (Ruggeri et al., 2024). In 2024, the United States produced 87.1 million pounds of hop, with production taking place primarily in the Pacific Northwest states of Washington (74% of production), Idaho (15%), and Oregon (11%) (USDA‐NASS, 2024).

Hop can be classified into three primary market classes: alpha or bittering, aroma, and dual‐purpose (Duarte et al., 2020). Bittering is contributed in the form of α‐acids, also known as humulones, and β‐acids, also known as lupulones. The α‐acids contribute most of the antibacterial and bittering components as they are isomerized during the wort boil of the brewing process into iso‐α‐acids (Steenackers et al., 2015). The β‐acids contribute to a lesser extent to the antibacterial and bittering components through oxidation during beer aging. High‐alpha hops (>14%) with high yields and improved disease resistance to the fungal disease hop powdery mildew (caused by the pathogen *Podosphaera macularis*) remain a critical objective in public hop breeding.

Hops also add aroma and flavor to beer through their essential oils, or total oil. The composition of hop total oils is complex and is influenced by the genetics of a specific hop cultivar, the environment, harvest timing, and storage conditions (Donner et al., 2020; Nesvadba et al., 2022). Hop total oils are composed of a variety of aromatic compounds, including geraniol, linalool, terpineol, terpinene, humulene, myrcene, and caryophyllene (Rutnik et al., 2022). The chemical pathways resulting in α‐acids, β‐acids, and total oil are complex and not completely understood (Mishra et al., 2018; Nagel et al., 2008; Patzak et al., 2021). In addition, the genetic diversity underlying the phenotypic variation of these traits remains poorly documented in hop. Biparental linkage mapping with restriction fragment length polymorphism, amplified fragment length polymorphisms, single sequence repeat, or Diversity Array Technology markers was used to identify markers associated with α‐acid content (Cerenak et al., 2009; Koie et al., 2005) and 50 sex, plant growth characteristics, and hop chemistry traits using association mapping (AM, McAdam et al., 2013). Several authors have also studied the inheritance and heritability of hop cone chemistry (J. Henning et al., 1997; McAdam et al., 2014); however, the use of markers in routine selection for chemical traits is limited in public sector breeding in the United States where selection is currently conducted primarily on a phenotypic basis.

The shape and size of hop cones can influence chemistry and harvestability. The α‐acids, β‐acids, and total oil are contained within lupulin and produced via the lupulin glands within hop cones. The total amount of α‐acids and β‐acids present within hop cones is correlated with the size and number of lupulin glands (Patzak et al., 2015, 2021). The concentration of α‐acids, β‐acids, and total oil can therefore be modified by the density of lupulin glands and overall size of hop cones to accommodate additional lupulin glands. Furthermore, while it is not extensively documented in the literature, hop cone size and shape can influence picking ability in commercial picking machines as well drying efficiency in the hop kilning process. Hop picking machines use a series of fans and dribble belts to mechanically separate cones from bines, leaves, and string. Cones must have adequate density to prevent yield loss from blowing out of the machine. Furthermore, they must have the ability to roll down belts without shattering. There is a widely understood anecdotal relationship between cones that are “open” or fluffy and cone shattering, though it has yet to be formally documented in the literature. Breeders tend to select for cones that are more closed in shape for these reasons. Efforts to phenotype and select upon these traits more thoroughly in US public hop breeding have been implemented using the HopBox imaging platform, which measures cone color and morphological traits and reads a QR code from images of hop cones (Altendorf et al., 2023).

Considering the desire to increase the content of α‐acids in hop, a method for high throughput screening is necessary for breeding programs to screen large populations. Near‐infrared spectroscopy (NIR) is a method commonly used in plant breeding to estimate quality and chemical components of plant samples in a fast, low‐cost, and nondestructive manner (Font et al., 2006). The method uses a model developed by correlating wavelength data from the near‐infrared region of the electromagnetic spectrum to actual lab‐generated chemical properties of the sample. The resulting model can then be used to predict values for unknown samples. NIR has been used in hop previously (Garden et al., 2000) primarily for varietal recognition (Fanning et al., 2025; Machado et al., 2020) and has been explored briefly for screening large populations for breeding (Killeen et al., 2014), though the technology has seen less use in breeding hop compared with other brewing ingredients, such as barley (Fox, 2020). NIR requires a small sample, which can be readily acquired by hand harvesting in hop. Standard methods for quantifying α‐acids, β‐acids, and total oil in hop require over 100 g of dried cones, which is less feasible to acquire by hand and may require pulling down and hauling bines to a stationary hop picker, which is considerably more time, labor, and infrastructure intensive and may not be possible for large‐scale mapping studies. Considering the potential gain in efficiency and the scale of phenotype data needed to reliably identify molecular markers through AM in hop, we sought to explore whether the method was adequate for obtaining reliable phenotype data.

The objectives of this study were to (1) assess the phenotypic variation within a large collection of female hop lines grown in Prosser, WA, over 2 years for α‐acid content, β‐acid content, total oil, and cone area and openness and make note of the reliability of NIR as a method for measuring chemistry traits; (2) conduct AM with the phenotypic data and SNP marker data derived from genotyping‐by‐sequencing (GBS) to identify marker trait associations; and (3) evaluate the potential gains from stacking beneficial alleles at identified markers for each trait. The study aims to pave the way for implementing GAB to improve the efficiency and precision of selection in an otherwise challenging perennial crop.

**Core Ideas**

- NIR is a rapid and cost‐effective method to measure cone chemistry traits in large populations of hop.
- Association mapping identified key loci involved in cone chemistry and morphology traits.
- The majority of significant markers exhibited a clear additive phenotype.
- Natural beneficial allele stacks are present within the breeding program that can be further exploited.
- Hop exhibits potential for the application of genomic selection.

## 2 MATERIALS AND METHODS

### 2.1 Germplasm acquisition, tissue collection, and field establishment

A collection of 1152 genotypes was acquired in the form of rhizomes or cuttings from a former Washington State University hop breeding program in Prosser, WA, the National Clonal Germplasm Repository, the USDA‐ARS hop breeding program in Corvallis, OR, and the Clean Plant Center Northwest in Prosser, WA. One mother plant from each genotype was established and used to propagate two replications using softwood cuttings. Rooted cuttings were up‐potted into 9‐cm square pots and hardened off outdoors for 2 weeks prior to planting in late June 2022. Transplants were established in a conventional US hop yard (4.6 m row spacing, 1 m plant spacing, and 5.5 m tall) but with two rows per conventional hop row, offset from either side of the poles by 0.5 m, and with plant positions offset from the adjacent row to maximize space use efficiency. The experiment was a randomized complete block design with two blocks surrounded by border rows of cv. Centennial (Kenny & Zimmermann, 1991). Each row of plants was irrigated with a single drip tube (Netafim; 2 L per h with 61 cm emitter spacing) and fertigated with 13.5 kg N per ha weekly from transplanting until bloom. Prophylactic imidacloprid, 1‐[(6‐chloro‐3‐pyridinyl)methyl]‐N‐nitro‐2‐imidazolidinimine, was applied once annually through the drip at 160 g a.i. per ha after training to prevent hop aphid (*Phorodon humuli*). A single strand of 45 kg tensile coir was strung at each plant position, and two bines were trained per plant. Carfentrazone‐ethyl 34.7 g a.i. per ha was applied for sucker management in mid‐April in 2023 and 2024, and again after training on July 4, 2023 and 2024. Trailing bines from neighboring genotypes were controlled by manually cutting plants apart using hedge shears to ensure the separation and integrity of the genotypes.

The population used in this experiment was previously described in Clare et al. (2024). Briefly, 50 mg of young leaf tissue was collected from each mother plant, frozen at −80°C, lyophilized, and sent to the Center for Qualitative Life Sciences at Oregon State University for extraction of genomic DNA using the Omega Biotek kit M1130 (MagBind Plant DNA DS). GBS libraries were developed for the population using the ApeKI enzyme (Elshire et al., 2011) and sequenced on an Illumina NextSeq 2000 with 96 samples per P2 cell. Reads were trimmed, quality filtered, and aligned to the Cascade reference genome (Padgitt‐Cobb et al., 2023) available on HopBase (Hill et al., 2017). Variant calling was conducted using HaplotypeCaller, GenotypeGVCF, and MergeVcfs in GATK 4.2.1.6 using a custom script (Clare et al., 2024). The variants were filtered using vcftools 0.1.16 (Danecek et al., 2011) for biallelic SNPs, minimum quality score of 30, allele depth of 3, 30% missing data for individuals and SNPs, minor allele frequency of 0.05, and data were imputed using Beagle 5.2 (Pook et al., 2020).

### 2.2 Phenotypic data collection

Harvest took place between late August and late September 2023 and 2024. Hop is typically harvested when the cones reach a certain optimal dry matter percentage specific to the cultivar. However, considering the scale of the experiment, the lack of information about the genotypes, and the range of harvest timings, harvest was conducted iteratively over the course of multiple weeks when cones were determined to be ready using subjective measures (aromatic with a tissue‐paper‐like consistency). Approximately 30 cones were hand‐harvested from each genotype and block. Cones were separated into two bags: (1) approximately 10 cones into a small sandwich‐size zipper lock plastic bag used to retain freshness, and (2) approximately 20 cones into a 1.36 kg leno mesh poly bags with drawcord (Glacier Valley Enterprises; Item No. 231). The 10 cone samples were imaged within 24 h of harvest along with a barcoded vertical feed slip‐on, tear‐off thermal tag (Loda Enterprises; SKU #440230) and color checker card using a previously described imaging platform for cones known as the HopBox (Altendorf et al., 2023). The HopBox pipeline outputs hop cone color values and dimensions (length, area, width, perimeter, and openness) for each cone within the images, and mass (optional, for the entire sample). Values were averaged for all cones within each image. Openness was calculated by dividing perimeter by cone length, and all values were converted to centimeters from pixels using the color checker card dimensions for reference. Considering that all morphological traits were highly correlated in our previous analyses of hop cones, only area and openness were utilized for this study (Altendorf et al., 2023). The 20 cone samples in mesh bags were placed into mesh laundry bags, buried among commercially grown and harvested hops in an American‐style hop kiln, and dried at 49°C–54°C for 8–12 h until they reached approximately 10% moisture. Sample bags were then placed in large mylar bags, flushed with nitrogen gas, vacuum sealed, and stored at −18°C until processing within 3 months. The 20 cone sample was ground using a 600 W personal blender (Nutribullet) and scanned three times each per the manufacturers’ instructions on a Europhins (Europhins QTA) NIR machine to measure α‐acids %, β‐acids %, and total oil (mL/100 g). The NIR utilized a partial least squares (PLS) linear regression from the Eurofins QTA database of dried hops spectra. The database comprises over 800 samples spanning multiple varieties and approximately 8 years of seasonal hops. Spectra were preprocessed using single normal variate standardization, and a spectral range from 4000 to 9000 cm^−1^ was used in the PLS model. The PLS model was created using Bruker Optics OPUS (version 8.5) PLS Toolbox. The average values from all three scans were calculated prior to downstream analyses. To validate the NIR method, we scanned 138 samples from our breeding program's advanced plots in 2024, which were submitted to a certified commercial laboratory for chemical analyses using high‐performance liquid chromatography (HPLC) (ASBC Method Hops‐14) and steam distillation (Hops‐13) to determine α‐acids %, β‐acids %, and total oil (mL/100 g). Pearson correlation was used between NIR‐ and laboratory‐derived methods to evaluate the validity of the NIR model and method.

### 2.3 Phenotypic analysis

Phenotypic data were tested for normality using shapiro.test() within Program R 4.3.0 and visual inspection of the histogram and quantile–quantile (*QQ*) plots (R Core Team, 2024). Datasets were analyzed using original (unaltered) data and normalized data using the boxcox() transformation. A linear model was used to calculate best linear unbiased estimates (BLUEs) for individual years where genotype was modeled as a fixed effect through one‐way analysis of variance using the base lm() command in R. BLUEs were calculated using the emmeans() function from the emmean 1.10.7 R package (Lenth, 2020). A linear mixed effects model was used to obtain best linear unbiased predictions (BLUPs) utilizing both years of data where genotypes and the genotypes by year interactions were modeled as random effects and the effect of year was modeled as a fixed effect using the lmer() command from the lme4 1.1‐35.1 R package (Bates et al., 2015). Broad‐sense heritability was calculated from the same linear mixed effects models used to calculate BLUPs, where the genetic variance is divided by the sum of genetic and residual variance. A Pearson's correlation matrix was calculated between BLUPs across traits.

### 2.4 Association mapping

Association mapping was conducted using the Bayesian Information and Linkage Disequilibrium Iteratively Nested Keyway (BLINK) of Genomic Association and Prediction Integrated Tool 3 (J. Wang & Zhang, 2021) using previously generated data. Analyses for BLINK models were conducted with zero and four principal components (PCs) as additional covariates to control for population structure. The number of PCs was determined using the total number that accounted for at least 25% of the genotypic variation. A total of 12 models for each trait were tested with traits including α‐acid content, β‐acid content, total oil content, cone area, and cone openness. A Bonferroni significance threshold of 5.62 was calculated at the 0.05 α level. Mean squared deviation (MSD) was calculated for each model and visual inspection of the *QQ* plots was performed for model selection.

### 2.5 Marker trait associations and gene identification

A genomic interval of 2 Mb (1 Mb upstream and downstream) was selected for candidate gene analysis. The LD() function from the gaston 1.6 R package was used to calculate pairwise *r* ^2^ between every marker pair per chromosome (Perdry et al., 2023). Linkage disequilibrium (LD) decay was calculated per chromosome using the loess function from R using marker comparisons within 10 Mb where *r* ^2^ was the response variable modeled by pairwise differences in physical distance. Candidate gene analysis was performed by extracting gene models and gene ontology terms of genes within identified intervals from the Cascade genome assembly (Padgitt‐Cobb et al., 2023). In addition, select gene models that did not contain gene ontology terms were translated to amino acids and basic local alignment search tool for proteins (BLASTp) was used to predict putative function (Altschul et al., 1997).

### 2.6 Phenotype by genotype and natural allele stacks

Significant markers identified via AM were plotted against the phenotype as phenotype by genotype jitter plots to determine the favorable allele. Subsequently, due to the number of significant markers and exponential number of genotypic classes that would be identified, markers exhibiting a clear effect on the phenotype were converted to a numeric value and summed, representing the total number of favorable alleles present within a genotype. These values were plotted against the BLUP phenotype as phenotype by favorable allele dosage plots and a line was fit using the geom_smooth linear model command. All figures were constructed using ggplot2 (Wickham, 2016).

## 3 RESULTS

### 3.1 NIR validation, phenotypic and genotypic data, and model optimization

**FIGURE 1:**
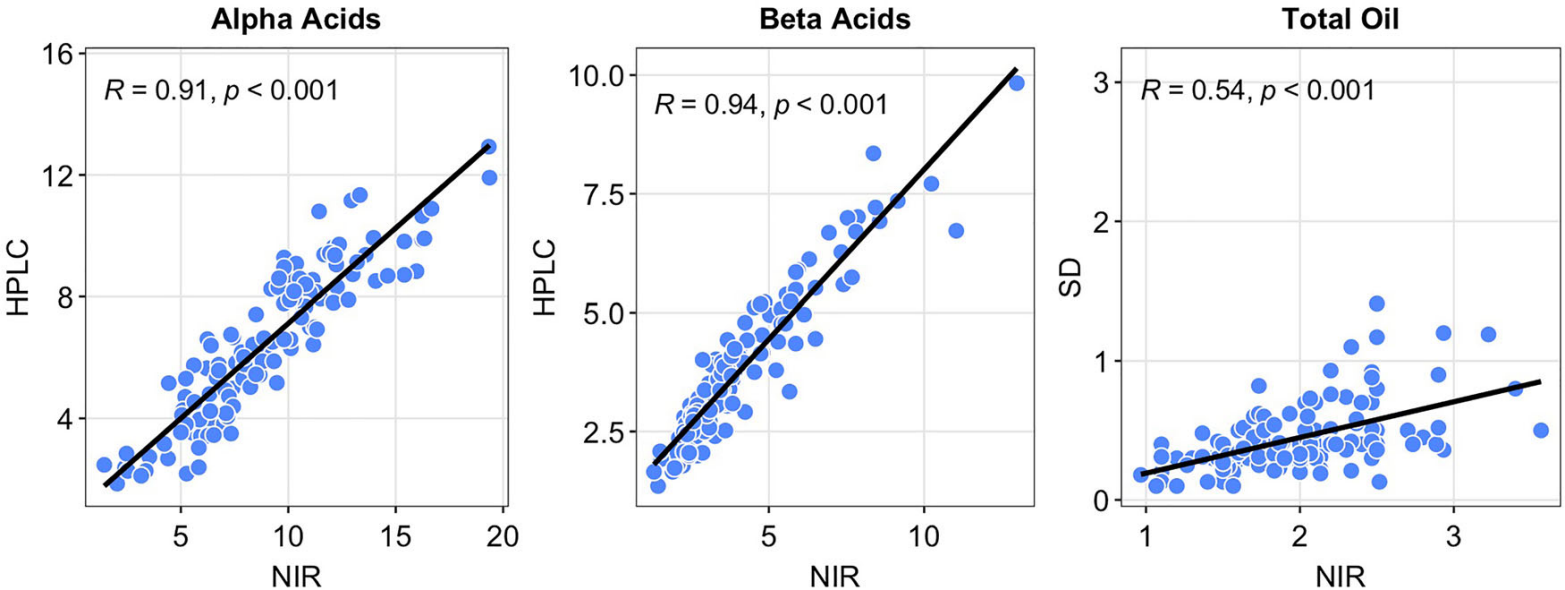
Validation of NIR‐derived values with high‐performance liquid chromatography (HPLC) using the ASBC Method Hops‐14 and steam distillation (SD)‐derived values using ASBC Method Hops‐13 from 138 samples from the hop breeding program in 2024.

To validate the NIR method, we compared phenotypic data from 138 hop genotypes collected as part of routine breeding activities using ASBC methods Hops‐14 (HPLC for α‐acids %, β‐acids %) and Hops‐13 (steam distillation for total oil content) to values derived from scans of the same samples on the NIR machine. All correlations were positive and significant (*p* < 0.001), with *R* = 0.54 for oil, 0.91 for α‐acids, and 0.94 for β‐acids (Figure 1). We previously validated cone morphological characteristics derived from the HopBox method (Altendorf et al., 2023).

Phenotypic data using the NIR and the HopBox was obtained for 790 genotypes. Genotypic data consisted of 20,861 biallelic SNPs. This dataset was previously described and was released for download via the National Center for Biotechnology Information (NCBI) (Clare et al., 2024; Supporting Information Files S1 and S2). The number of genotypes was reduced to 529 for AM to obtain overlap between the phenotypic and genotypic datasets (Supporting Information File S3). A total of 461 and 514 genotypes were used for 2023 and 2024 BLUEs, respectively. Phenotypic datasets were determined to not satisfy the assumption of having a normal distribution. However, the datasets did not display extreme skewness and were therefore analyzed both unaltered and transformed before generating BLUEs and BLUPs. Variation of phenotypic values in the population for all traits, calculated as BLUPs, is shown in Figure 1, and broad‐sense heritability estimates are reported in each facet. These values ranged from 0.32 for cone area (least heritable), to 0.71 for α‐acids (most heritable). Variance component estimates for each trait are reported in Table S1. There were several significant (*α* = 0.01) correlations between BLUPs, including a positive relationship between α‐acids and total oil (*R* = 0.55) as well as β‐acids and total oil (*R* = 0.24) (Figure S1). There was a negative correlation between total oil and cone openness and α‐acids (*R* = −0.23 and −0.15).

To optimize the model quality for AM analyses, we tested the inclusion of four PCs and transformed versus unaltered data. Variable outcomes were observed from visually inspecting the resulting *QQ* plots, depending on the trait (Figure S2). For example, in some cases, inclusion of PCs resulted in improved model quality, and in others it was reduced. The result was similar for data transformation (Figure S2). These visual differences were not evident from MSD calculations due to varying maximum −log10 *p* values and marginal improvements (Table S2). Due to these discrepancies and inability to identify an optimal model, we ran every possible model combination for each trait (i.e., BLUPs across years, BLUEs for each year, transformed and unaltered) and further investigated markers that were significant in at least 25% (3 out of 12) models. In most cases, models utilizing transformed data, inclusion of PCs, or both identified few additional regions that contained candidate genes with a compelling rationale to be involved with the trait.

### 3.2 Significant intervals

A total of 49 significant markers were identified using the 25% criterion described above, with 43 unique markers across all traits (Table 1). Chromosome X and chromosome 4 contained the highest number of significant markers, whereas chromosome 9 contained the fewest (Table 1). A total of 15, 10, 7, 7, and 10 significant markers were identified for α‐acids, β‐acids, total oil, cone area, and cone openness (Table 1). Significant markers were distributed throughout the genome for α‐acids, β‐acids, cone area, and cone openness, whereas significant markers for total oil were predominantly identified on chromosome X (Table 1). Manhattan plots for each trait, including all significant markers across all models tested, are shown in Figures 3, 4, 5, 6, 7.

**TABLE 1:** Significant marker distribution across the hop genome. The total number of markers for a chromosome may exceed the total number of unique markers due to pleiotropic markers.

Chromosome	α‐acid	β‐acid	Total oil	Area	Openness	Total (unique)
1				2	1	3
2	3				2	5
3	2	1			1	4
4	4	4			1	9 (7)
5				2	1	3
6	1				2	3
7	1	1			1	3 (2)
8	1	1	1	2	1	6 (5)
9	1			1		2
X	2	3	6			11 (9)
Genome	15	10	7	7	10	43

### 3.3 Pleiotropic markers

Pleiotropic markers were defined as the same significant markers identified for more than one trait. A total of six markers were identified across two traits, primarily involving chemical traits (Table 2). No marker was identified in more than two traits. In four of the cases, the marker was only identified in one model for the second trait indicating this may be a false positive or that the locus is involved to a lesser extent with the secondary trait.

**TABLE 2:** List of significant markers identified via association mapping. Markers follow the naming convention of chromosome followed by physical position (bp) on the Cascade reference assembly (Padgitt‐Cobb et al., 2023). The traits and number of models the marker was identified in are displayed, along with the maximum logarithm of odds (LOD) and effect is reported. The number of genes and the top candidate gene and function with the genomic interval are also reported.

Marker^a^	Trait(s)	Models	Max LOD	Max effect	MAF	Candidates	Top candidate gene	Putative function
Chr4_341967019	α‐acid, β‐acid	7, 7	13.6, 15.3	1.09, 0.56	0.43	25	HUMLU_CAS0039836.t1.p1	2‐Methylpropanoate—CoA ligase
Chr8_204116007	α‐acid, Area	1, 5	14.7, 8.6	0.63, 0.55	0.12	7	HUMLU_CAS0068615.t1.p1	Tify domain binding domain
ChrX_193884342	α‐acid, Total Oil	5, 1	18.5, 4.8	1.46, 0.13	0.33	18	HUMLU_CAS0023276.t1.p1	Glycosyl hydrolase family 9
Chr4_340839925	α‐acid, β‐acid	1, 4	7.6, 16.6	0.31, 0.69	0.33	29	HUMLU_CAS0039797.t1.p1	Adrenodoxin‐like protein
ChrX_214567482	α‐acid, Total Oil	2, 2	17.1, 19.7	1.47, 0.28	0.35	319	HUMLU_CAS0023895.t1.p1	Cinnamoyl‐CoA reductase 1‐like
Chr7_2345310	α‐acid, β‐acid	2, 1	8.3, 5.9	1.13, 0.41	0.15	39	HUMLU_CAS0052907.t1.p1	3‐Hydroxyisobutyryl‐CoA hydrolase
Chr2_94993080	α‐acid	8	14.2	3.17	0.04	7	HUMLU_CAS0014134.t1.p1	Enoyl‐(Acyl carrier protein) reductase
Chr3_259895702	α‐acid	7	12.7	1.37	0.25	9	HUMLU_CAS0031934.t1.p1	Cyclic dof factor 3
Chr2_389144853	α‐acid	6	18.2	2.48	0.11	17	HUMLU_CAS0018918.t1.p1	Pyruvate dehydrogenase
Chr2_52775593	α‐acid	4	12.4	1.40	0.34	8	HUMLU_CAS0013523.t1.p1	Alpha, alpha‐trehalose‐phosphate synthase
Chr9_143875744	α‐acid	4	9.7	2.03	0.05	12	HUMLU_CAS0072734.t1.p1	Helicase
Chr3_22216642	α‐acid	3	7.2	1.17	0.22	33	HUMLU_CAS0028152.t1.p1	3‐Hydroxyisobutyrate dehydrogenase
Chr4_201164213	α‐acid	3	8.2	0.75	0.37	1	HUMLU_CAS0038156.t1.p1	Autophagy‐related protein
Chr4_342828442	α‐acid	3	13.3	1.34	0.23	50	HUMLU_CAS0039862.t1.p1	UDP‐glucoronosyl/UDP‐glucosyl transferase
Chr6_313334984	α‐acid	3	7.8	1.04	0.17	60	HUMLU_CAS0052610.t1.p1	UbiA prenyltransferase
ChrX_244034803	β‐acid	7	17.2	0.87	0.38	4	HUMLU_CAS0024160.t1.p1	Myrcene synthase
Chr4_26989754	β‐acid	7	13.0	0.69	0.13	8	HUMLU_CAS0036405.t1.p1	Acyl‐coenzyme A oxidase
Chr4_16779968	β‐acid	4	8.9	0.82	0.09	76	HUMLU_CAS0036078.t1.p1	2‐Keto‐3‐deoxy‐L‐rhamnonate aldolase
Chr8_40403670	β‐acid	4	10.2	0.42	0.41	50	HUMLU_CAS0062960.t1.p1	Cytochrome P450
ChrX_413717679	β‐acid	4	19.5	0.55	0.45	34	HUMLU_CAS0026425.t1.p1	Ras‐related protein
Chr3_148491125	β‐acid	3	18.9	0.89	0.26	4	HUMLU_CAS0030446.t1.p1	BRO1‐like domain
ChrX_405160052	β‐acid	3	8.4	0.47	0.12	11	HUMLU_CAS0026204.t1.p1	Fructose‐1‐6‐bisphosphatase
ChrX_121312918	Total Oil	6	7.6	0.21	0.35	13	HUMLU_CAS0022354.t1.p1	NAD(P)H‐quinone oxidoreductase
Chr8_229402574	Total Oil	4	14.9	0.36	0.09	9	HUMLU_CAS0068949.t1.p1	Serine carboxypeptidase
ChrX_414804061	Total Oil	4	8.0	0.20	0.12	31	HUMLU_CAS0026479.t1.p1	Regulator of Vps4 in the MVB pathway
ChrX_192570590	Total Oil	3	9.9	0.35	0.08	10	HUMLU_CAS0023249.t1.p1	Acetyl‐CoA carboxylase
ChrX_214321371	Total Oil	3	10.9	0.24	0.46	319	HUMLU_CAS0023894.t1.p1	Hydroxymethylglutaryl‐coA reductase
Chr5_306280798	Area	7	12.3	0.47	0.19	52	HUMLU_CAS0045310.t1.p1	RGS1‐HXK1‐interacting protein
Chr1_434468125	Area	4	10.2	0.54	0.50	10	HUMLU_CAS0009337.t1.p1	Nitrogen regulatory protein
Chr5_105190810	Area	4	8.6	0.80	0.09	52	HUMLU_CAS0045310.t1.p1	RGS1‐HXK1‐interacting protein
Chr1_422531025	Area	3	12.1	0.31	0.39	28	HUMLU_CAS0008962.t1.p1	Peptidyl‐prolyl cis‐trans isomerase
Chr8_67399361	Area	3	10.4	1.24	0.07	37	HUMLU_CAS0064171.t1.p1	Subtilisin‐like protease
Chr9_8295045	Area	3	11.5	0.74	0.05	30	HUMLU_CAS0070528.t1.p1	Nuclear intron maturase
Chr4_18198833	Openness	6	9.4	0.08	0.34	79	HUMLU_CAS0036197.t1.p1	Cinnamyl alcohol dehydrogenase
Chr5_16477979	Openness	6	17.2	0.09	0.24	24	HUMLU_CAS0040467.t1.p1	Alpha, alpha‐trehalose‐phosphate synthase
Chr8_40129263	Openness	6	17.1	0.26	0.09	49	HUMLU_CAS0062943.t1.p1	Endoglucanase
Chr6_299269481	Openness	5	6.8	0.09	0.17	25	HUMLU_CAS0051971.t1.p1	Tryptophan decarboxylase
Chr3_95899604	Openness	4	10.5	0.10	0.18	6	HUMLU_CAS0029797.t1.p1	E3 ubiquitin‐protein ligase
Chr1_136782373	Openness	3	7.7	0.10	0.15	3	HUMLU_CAS0003887.t4.p1	Bifunctional 3‐dehydroquinate dehydratase/shikimate dehydrogenase
Chr2_27433056	Openness	3	10.8	0.08	0.42	19	HUMLU_CAS0012850.t1.p1	Lignin‐forming anionic peroxidase
Chr2_408675615	Openness	3	9.6	0.13	0.11	46	HUMLU_CAS0019726.t1.p1	Allene oxide cyclase
Chr6_179085251	Openness	3	9.9	0.13	0.08	9	HUMLU_CAS0050503.t1.p1	Zinc finger CCCH domain‐containing protein
Chr7_208799781	Openness	3	9.1	0.11	0.20	11	HUMLU_CAS0057246.t1.p1	Transcription factor IBH1

*Note*: Effect and MAF (minor allele frequency) rounded to two decimals.

^a^ Marker nomenclature consists of chromosome and position in base pairs (bp).

### 3.4 Genomic hotspots

Unlike pleiotropy where the same marker was identified for multiple traits, genomic hotspots were identified as regions with multiple significant markers in proximity with others for the same or multiple traits. Six hotspots were identified across the hop genome for the chemistry and morphological traits measured with two on chromosome 4, one on chromosome 8, and three on chromosome X (Table 3). The genomic hotspots clustered predominantly as cone chemistry traits with cone openness (α‐acid, β‐acid, and total oil as combinations), whereas markers associated with cone area were featured in a unique hotspot.

**TABLE 3:** Genomic hotspots identified within proximity of the hop genome. The significant markers (chromosome followed by base pair [bp] position), corresponding trait(s), and the interval size in bp are provided.

Genomic hotspot	Markers	Trait(s)	Interval size (bp)
Chr4_GHS1	Chr4_16779968	β‐acid	1,418,865
	Chr4_18198833	Openness	
Chr4_GHS2	Chr4_340839925	β‐acid	1,988,517
	Chr4_341967019	α‐acid/β‐acid	
	Chr4_342828442	α‐acid	
Chr8_GHS3	Chr8_40129263	Openness	274,407
	Chr8_40403670	β‐acid	
ChrX_GHS4	ChrX_192570590	Total oil	21,996,892
	ChrX_193884342	α‐acid/total oil	
	ChrX_214262650	Total oil	
	ChrX_214321371	Total oil	
	ChrX_214567482	α‐acid/total oil	
ChrX_GHS5	ChrX_405160052*	β‐acid	9,644,009*
	ChrX_413717679	β‐acid	1,086,382
	ChrX_414804061	Total oil	
ChrX_GHS6	ChrX_422531025	Area	11,937,100
	ChrX_434468125	Area	

*Note*: Astericks following the maker name indicates a lower confidence inclusion, with the corresponding interval size reflecting the size in bp should the marker be included.

### 3.5 Candidate gene identification

LD decay was calculated at approximately 1.1 Mb from half the maximum, when averaged across each chromosome (Figure S3). LD decay did not decrease below a 0.1 *r* ^2^ threshold for any chromosome, except for chromosome X (Figure S3). As such, the candidate gene identification window was set at 2.0 Mb. A total of 1210 candidate genes were identified across all significant markers and traits, with the number of candidate genes within the 2 Mb window ranging from 1 to 319 (Table 2). The largest number of candidate genes were identified in α‐acid with 635 candidate genes. This was followed by total oil, β‐acid, cone openness, and cone area with 400, 280, 271, and 164 candidate genes, respectively. However, over 319 candidate genes were included in α‐acid and total oil from the ChrX_GHS4 genomic hotspot (Table 3). Additionally, α‐acid had the highest number of loci and was involved in all pleiotropic markers.

### 3.6 Natural allele stacks

Significant markers identified in at least three models and exhibiting a clear favorable allele on the phenotype were included in the analysis of natural allele stacks. In total, ten, nine, seven, six, and nine were utilized for α‐acid, β‐acid, total oil, cone area, and cone openness (Figures 3, 4, 5, 6, 7). In all traits, genotypes within the population naturally carrying increasing numbers of favorable alleles exhibited a favorable change in the phenotypic value for all traits (increase for α‐acid, β‐acid, total oil, and cone area and decrease in cone openness) with Pearson correlation *R* values of 0.70, 0.63, 0.53, 0.46, and −0.52 (Figures 3, 4, 5, 6, 7).

## 4 DISCUSSION

GAB has the potential to greatly improve efficiency in hop breeding. However, knowledge of the genetic control of important traits is needed. Efforts to identify marker trait associations in hop have been largely focused on disease resistance (Havill et al., 2023; J. A. Henning et al., 2011; Olatoye et al., 2023) and plant sex (Čerenak et al., 2019; Clare et al., 2024). Quality traits, such as α‐acids, β‐acids, and total oil, can be challenging to phenotype in large populations due to laboratory costs and sample volume requirements. Likely, as a result, only few studies report on genetic mapping of quality traits, including bi‐parental mapping populations (Cerenak et al., 2009; Koie et al., 2005; McAdam et al., 2013) and one other AM study using historical breeding program data with 116 individuals and UNEAK markers from TASSEL (J. Henning et al., 2019). Here, we report the largest scale (in terms of individuals) AM for quality traits in hop utilizing the latest version of the hop genome (Padgitt‐Cobb et al., 2023) and the first to employ high‐throughput phenotyping techniques, including NIR to rapidly screen for chemical traits and the HopBox, which utilized image analyses to determine cone morphological characteristics (Altendorf et al., 2023). Our objectives were to characterize a large population of female hop plants for these traits, evaluate the NIR method for obtaining phenotype data on a large population, identify markers, and evaluate the potential gains from stacking beneficial alleles utilizing existing genotypes within our collection.

**FIGURE 2:**
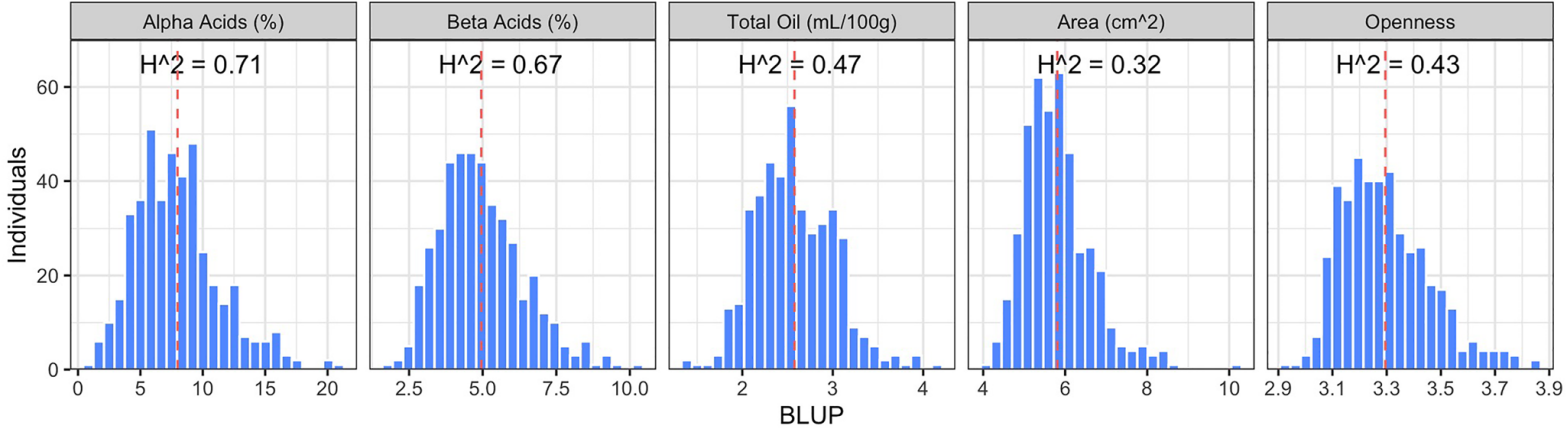
Histograms of best linear unbiased predictions (BLUPs) calculated from phenotypic data across 529 hop genotypes for α‐acids (%), β‐acids (%), total oil (mL/100 g), cone area (cm^2^), and cone openness (perimeter/length). Vertical red dashed lines represent the mean of all individuals, and broad‐sense heritability estimates for each trait are featured in the corresponding facets.

The population of 529 female hop plants utilized herein showed significant variation for the traits evaluated with higher broad‐sense heritability for chemical traits (*H* ^2^ = 0.47–0.71) compared with cone morphological traits (0.32 and 0.43) (Figure 2). Chemical traits are known to be highly heritable in hop, especially α‐acids. For example, J. Henning et al. (2019) reported broad‐sense estimates of 0.85 for α‐acids, 0.73 for β‐acids, and 0.61 for total oil, and J. Henning et al. (1997) reported narrow sense estimates of 0.88 ± 0.17 for α‐acids, 0.35 ± 0.02 for β‐acid, and 0.12 ± 5.3 × 10^−4^ for total oil. These results are also supported by Beatson (2005)’s estimates of heritability for α‐acids using both parent‐offspring regression (*h* ^2^ = 0.69) and single plant estimates (0.98). Previous estimates of broad‐sense heritability for cone morphology traits were previously reported from a smaller set of genotypes and ranged from 0.23 for cone length to a higher estimate of 0.59 for cone openness (Altendorf et al., 2023). While McAdam et al. (2014) did not measure cone morphological traits per se, in their study, plant growth traits generally also had lower narrow‐sense heritability estimates when compared with cone chemistry traits. Overall, our results combined with others support the idea that the genetic variance associated with cone chemical traits in hop is large relative to environment or other error components.

In addition to the traits exhibiting high heritability and repeatability, several of the top candidate genes identified within the 2.0 Mb window surrounding the significant markers had logical predicted functions (Table 2). For example, multiple significant markers associated with α‐acid content were nearby candidate genes that potentially function directly within the α‐acid pathway. These include some of the highest effect markers identified in this study, such as an enoyl‐(acyl carrier protein) reductase (Chr2_94993080), a pyruvate dehydrogenase (Chr2_389144853), a 3‐hydroxyisobutyrate dehydrogenase (Chr3_22216642), and a 3‐hydroxyisobutyryl‐CoA hydrolase (Chr7_2345310) (Table 2). The same is true for β‐acid, with the identification of genes involved directly within the β‐acid pathway, such as a 2‐keto‐3‐deoxy‐L‐rhamnonate aldolase (Chr4_16779968) and an acyl‐coenzyme A oxidase (Chr4_26989754). All of these genes are vital for the production or availability of precursors required for α‐acid and β‐acid production (Arent et al., 2008; Hernández et al., 2017). Furthermore, the top candidate gene identified in both α‐acid and β‐acid, is predicted to encode a 2‐methylpropanoate—CoA ligase (Chr4_341967019), vital to produce 3‐hydroxyisobutyryl‐CoA, a precursor and therefore a rate‐limiting component in the production of α‐ and β‐acid (Xu et al., 2013). Other significant markers are potentially involved indirectly in α‐acid and β‐acid production through pathway regulation, floral transition, and chemical export through genes such as a cyclic dof factor 3 (Chr3_259895702), an alpha, alpha‐trehalose‐phosphate synthase (Chr2_52775593), an autophagy‐related protein (Chr4_201164213), a Ras‐related protein (ChrX_413717679), and a BRO1‐related domain (Chr3_148491125; Table 2) (Corrales et al., 2017; Crespo & León, 2000; Kim et al., 2005).

For total oil, the top gene candidate is predicted to encode a cinnamoyl‐CoA reductase 1‐like (ChrX_214567482) and is known to be a key enzyme in lignin through the reduction of cinnamoyl‐CoA esters into cinnamaldehyde, which is responsible for the cinnamon aroma and flavor (Kawasaki et al., 2005; Liu et al., 2021). Serine carboxypeptidase (SCP, Chr8_229402574) identified for total oil is not predicted to directly influence the production of total oil, but SCPs are known to be vital in protein catabolism, which could activate or deactivate pathways (Breddam, 1986). Hydroxymethylglutaryl‐CoA reductase (ChrX_214321371) is a key enzyme in the mevalonate pathway and therefore a rate‐limiting enzyme for the production of terpene and flavonoid precursor dimethylallyl diphosphate, which in turn is utilized for the production of compounds such as myrcene, caryophyllene, humulene, and xanthohumol (Chen et al., 2023; Lange et al., 2000; Nagel et al., 2008; G. Wang et al., 2008; Yang et al., 2024). Acetyl‐CoA carboxylase (ChrX_192570590) is critical in the production of the precursor malonyl‐CoA for the xanthohumol pathway (Y. Wang et al., 2022; Yang et al., 2024).

All candidate genes identified for cone area are implicated in plant development. For example, an RGS1‐HXK1‐interacting protein (Chr5_105190810 and Chr5_306280798) is predicted to function in seedling development through sugar signaling and pathogen defense (Chen et al., 2023; Tang et al., 2023; Y. Wang et al., 2022). Similarly, subtilisin‐like proteases (Chr8_67399361) are known to play a critical role in plant growth (Figueir et al., 2014). Multiple candidate genes for cone openness have implications in structural molecule synthesis, such as cinnamyl alcohol dehydrogenase (Chr4_18198833), an endoglucanase (Chr8_40129263), and lignin‐forming anionic peroxidase (Chr2_27433056), which are involved in lignin and cellulose production (Li et al., 2022; Cosgrove, 2005). Both tryptophan decarboxylase (Chr6_299269481) and allene oxide cyclase (Chr2_408675615) are involved in hormone synthesis that could influence hop development (De Luca & St Pierre, 2000; Yang et al., 2023). Regions that contain no obvious gene responsible for the trait could be due to incomplete or unannotated gene models, hypothetical/uncharacterized proteins, genes that indirectly influence the pathway, for example, transcription factors or linkage. The top candidates reported (Table 2) are only predictions and will require validation to determine if the selected gene is causal. Additionally, further analysis of all genes that lacked gene ontology, reannotation of the Cascade reference genome, or extension to a hop pangenome for comparative genomics of structural and presence/absence variation could uncover further candidate genes.

No single large‐effect marker was detected for any of the traits tested, and, in many cases, significant markers were identified across the genome, suggesting both cone chemistry and morphology in hop are highly quantitative in nature. Chromosome 4 appears to be important for both cone chemistry and openness, and chromosome X for chemistry, especially oil content (Tables 1 and 3). This finding aligns with previous mapping work where markers for α‐acids were small‐effect and numerous (Henning et al., 2019), and inherited in a quantitative manner (Cerenak et al., 2009). Since many of these total oil loci appear to be on chromosome X, this could be a result of dosage, whereby male plants have one copy and females have two copies, or different hop lineages bringing together distinct X haplotypes. For example, admixture between North American and European hop backgrounds may have brought together distinct haplotypes on chromosome X, resulting in the observed signal.

**FIGURE 3:**
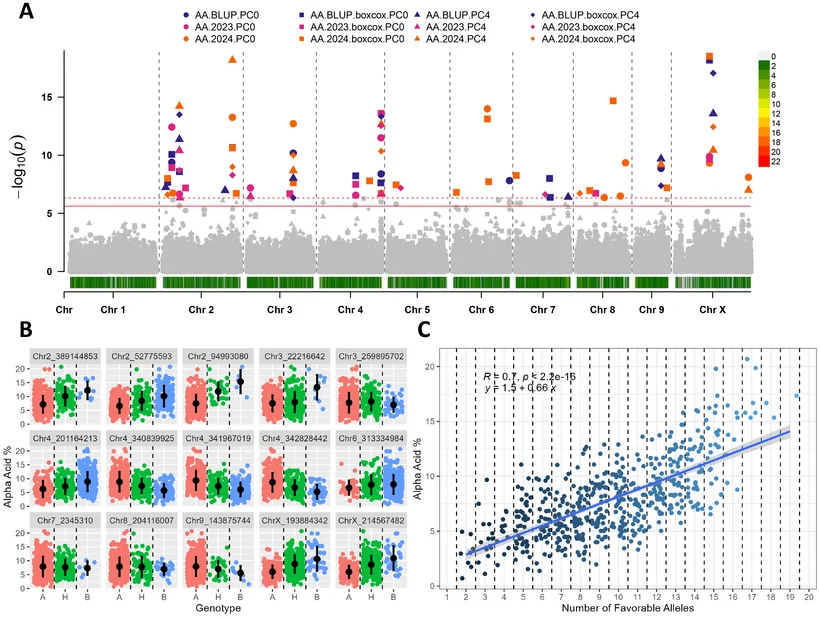
Results from association mapping for α‐acid content in hop. (A) Manhattan plot of α‐acid content association mapping. Zero principal components (PCs), zero PCs with boxcox normalization, four PCs, and four PCs with boxcox normalization are shown as circles, squares, triangles, and diamonds, respectively. Combined best linear unbiased predictions (BLUPs) and best linear unbiased estimates (BLUEs) for 2023 and 2024 subsets are shown in purple, pink, and orange, respectively. The logarithm of odds (LOD) score is shown on the *y*‐axis, along with the 0.01 and 0.05 LOD thresholds as dashed and solid red lines, respectively. Chromosomes are shown on the *x*‐axis along with marker density. (B) Phenotype by genotype jitter plots of significant markers identified via association mapping in panel A. Homozygous reference (A), heterozygous (H), and homozygous alternative (B) alleles are shown in red, green, and blue, respectively, on the *x*‐axis. The α‐acid content is shown on the *y*‐axis. The mean and standard deviation are also shown as a black dot and line, respectively. (C) Favorable allele dosage by phenotype jitter plots of markers with evident additive effect identified in panel B. The number of favorable alleles is on the *x*‐axis, and α‐acid content is shown on the *y*‐axis.

**FIGURE 4:**
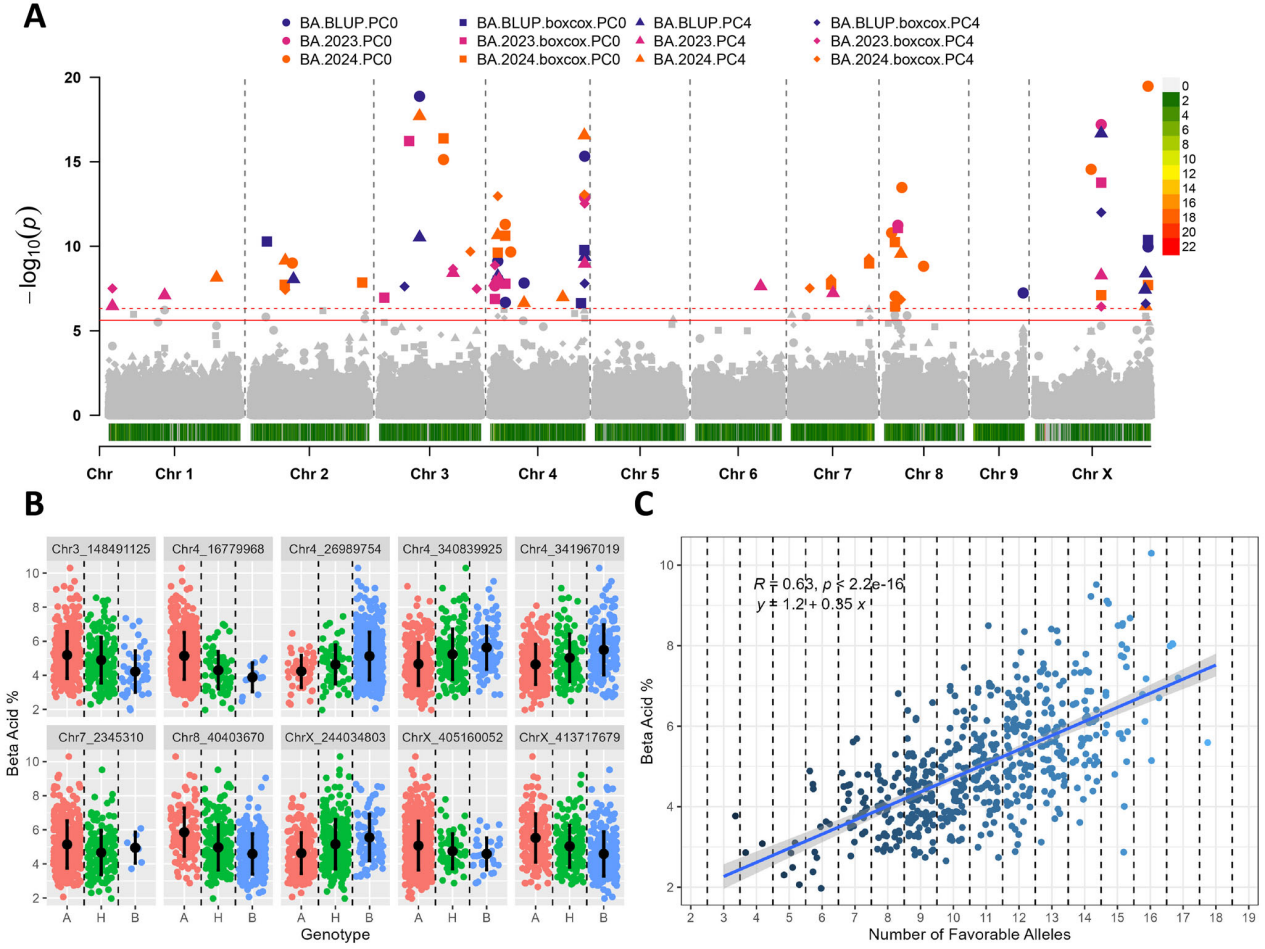
Results from association mapping for β‐acid content in hop. (A) Manhattan plot of β‐acid content association mapping. Zero principal components (PCs), zero PCs with boxcox normalization, four PCs, and four PCs with boxcox normalization are shown as circles, squares, triangles, and diamonds, respectively. Combined best linear unbiased predictions (BLUPs) and best linear unbiased estimates (BLUEs) for 2023 and 2024 subsets are shown in purple, pink, and orange, respectively. The logarithm of odds (LOD) score is shown on the *y*‐axis, along with the 0.01 and 0.05 LOD thresholds as dashed and solid red lines, respectively. Chromosomes are shown on the *x*‐axis along with marker density. (B) Phenotype by genotype jitter plots of significant markers identified via association mapping in panel A. Homozygous reference (A), heterozygous (H), and homozygous alternative (B) alleles are shown in red, green, and blue, respectively, on the *x*‐axis. The β‐acid content is shown on the *y*‐axis. The mean and standard deviation are also shown as a black dot and line, respectively. (C) Favorable allele dosage by phenotype jitter plots of markers with evident additive effect identified in panel B. The number of favorable alleles is on the *x*‐axis, and β‐acid content is shown on the *y*‐axis.

**FIGURE 5:**
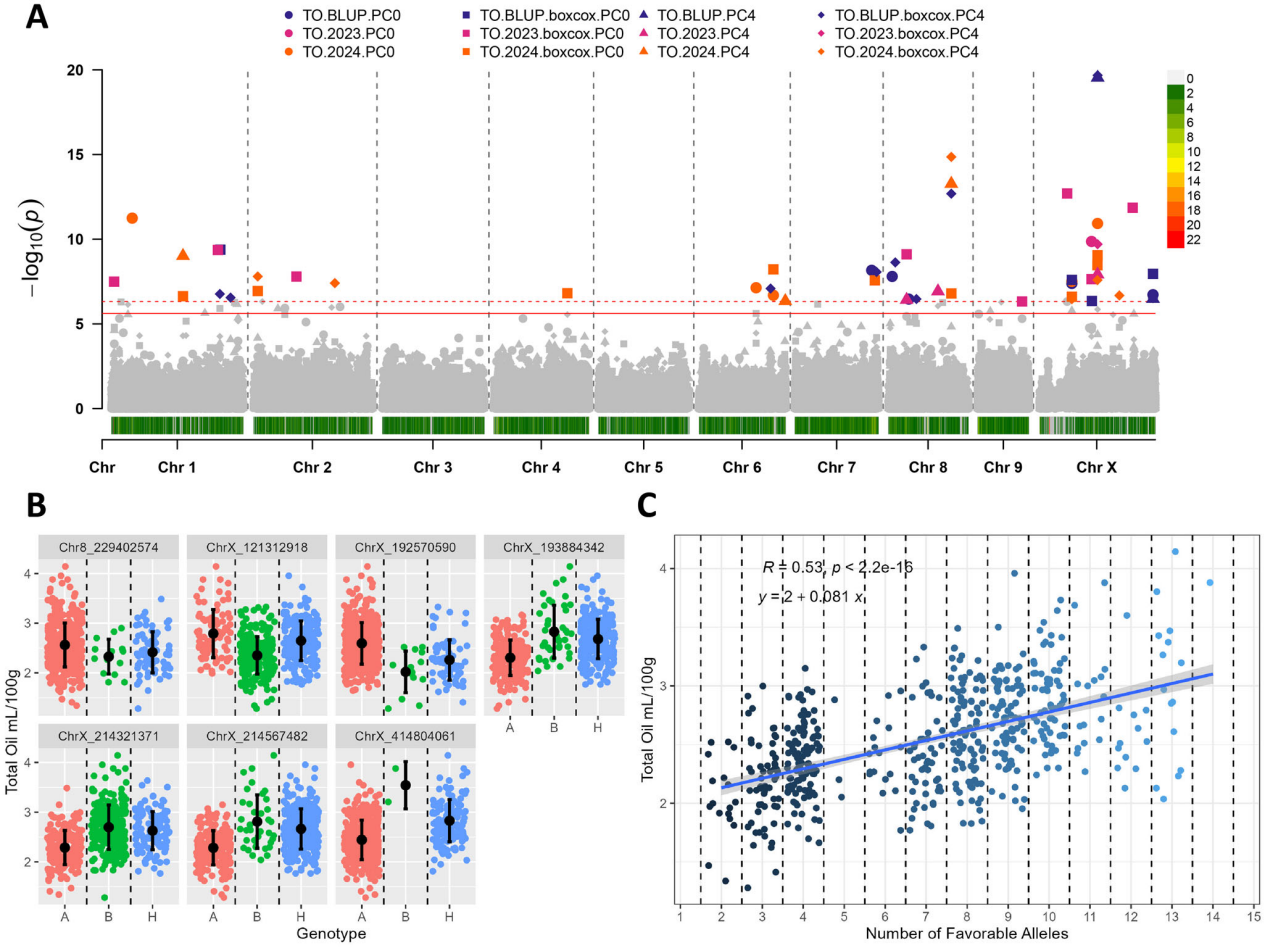
Results from association mapping of total oil in hop. (A) Manhattan plot of oil content association mapping. Zero principal components (PCs), zero PCs with boxcox normalization, four PCs, and four PCs with boxcox normalization are shown as circles, squares, triangles, and diamonds, respectively. Combined best linear unbiased predictions (BLUPs) and best linear unbiased estimates (BLUEs) for 2023 and 2024 subsets are shown in purple, pink, and orange, respectively. The logarithm of odds (LOD) score is shown on the *y*‐axis, along with the 0.01 and 0.05 LOD thresholds as dashed and solid red lines, respectively. Chromosomes are shown on the *x*‐axis along with marker density. (B) Phenotype by genotype jitter plots of significant markers identified via association mapping in panel A. Homozygous reference (A), heterozygous (H), and homozygous alternative (B) alleles are shown in red, green, and blue, respectively, on the *x*‐axis. The total oil content is shown on the *y*‐axis. The mean and standard deviation are also shown as a black dot and line, respectively. (C) Favorable allele dosage by phenotype jitter plots of markers with evident additive effect identified in panel B. The number of favorable alleles is on the *x*‐axis, and total oil content is shown on the *y*‐axis.

**FIGURE 6:**
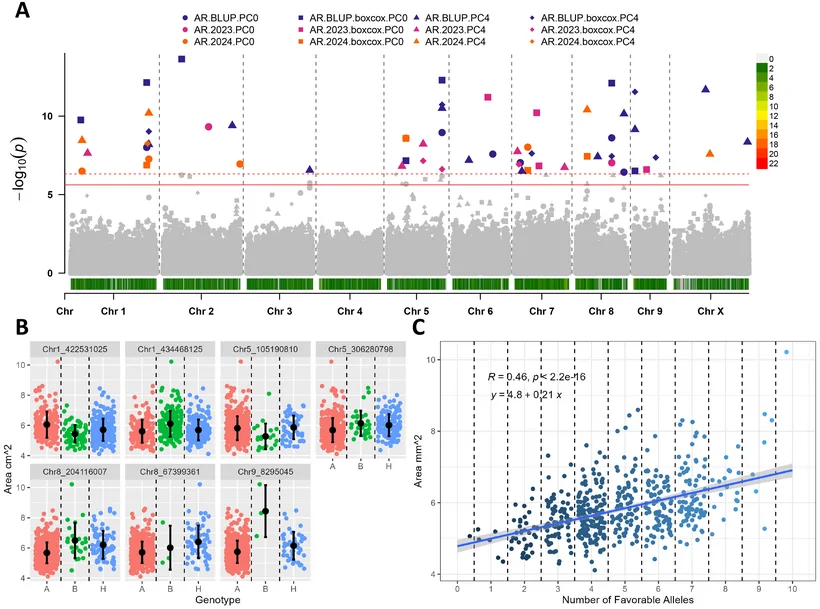
Results from association mapping of cone area in hop. (A) Manhattan plot of cone area content association mapping. Zero principal components (PCs), zero PCs with boxcox normalization, four PCs, and four PCs with boxcox normalization are shown as circles, squares, triangles, and diamonds, respectively. Combined best linear unbiased predictions (BLUPs) and best linear unbiased estimates (BLUEs) for 2023 and 2024 subsets are shown in purple, pink, and orange, respectively. The logarithm of odds (LOD) score is shown on the *y*‐axis, along with the 0.01 and 0.05 LOD thresholds as dashed and solid red lines, respectively. Chromosomes are shown on the *x*‐axis along with marker density. (B) Phenotype by genotype jitter plots of significant markers identified via association mapping in panel A. Homozygous reference (A), heterozygous (H), and homozygous alternative (B) alleles are shown in red, green, and blue, respectively, on the *x*‐axis. The cone area content is shown on the *y*‐axis. The mean and standard deviation are also shown as a black dot and line, respectively. (C) Favorable allele dosage by phenotype jitter plots of markers with evident additive effect identified in panel B. The number of favorable alleles is on the *x*‐axis, and cone area content is shown on the *y*‐axis.

**FIGURE 7:**
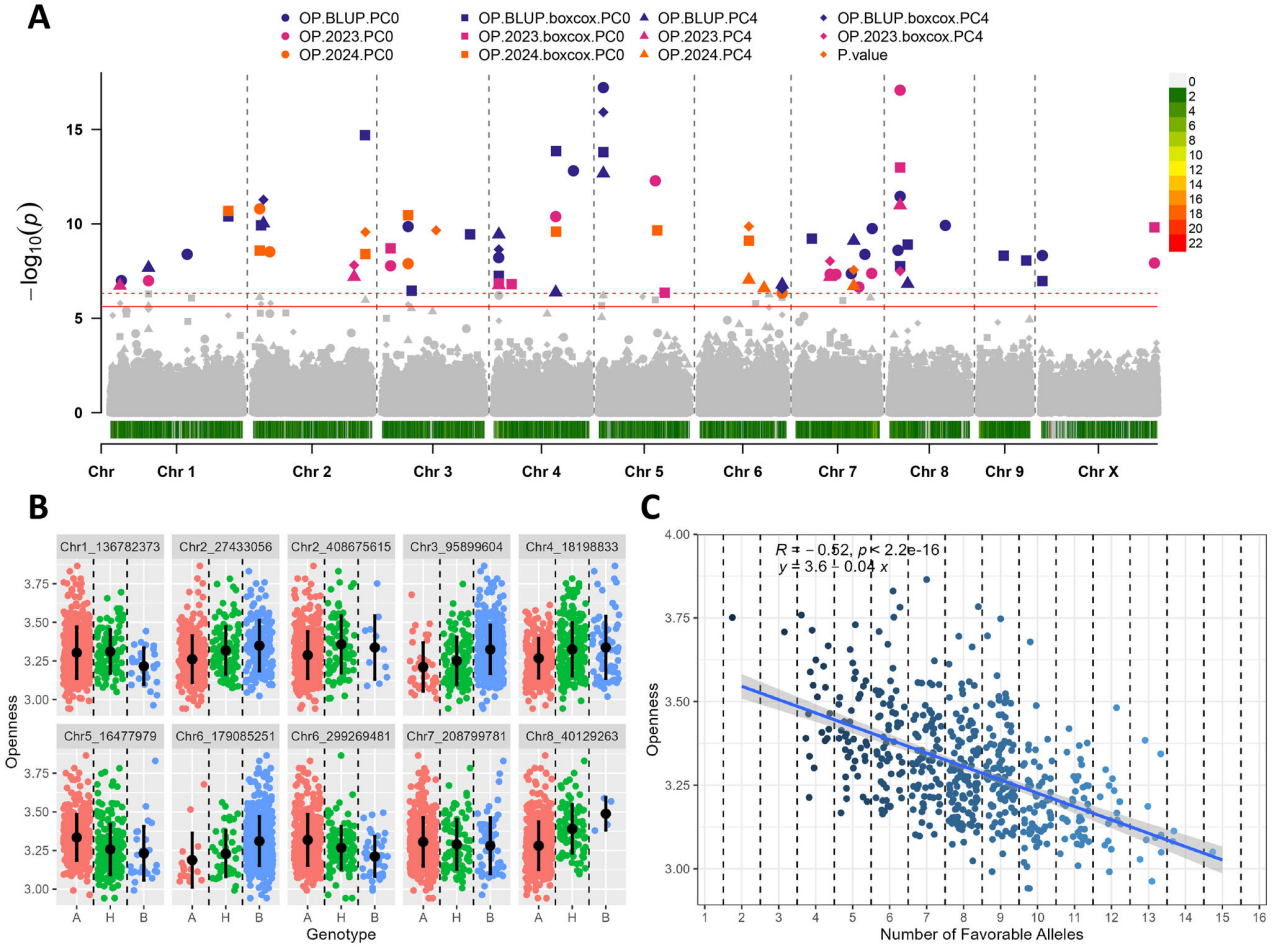
Results from association mapping of cone openness in hop. (A) Manhattan plot of cone area association mapping. Zero principal components (PCs), zero PCs with boxcox normalization, four PCs, and four PCs with boxcox normalization are shown as circles, squares, triangles, and diamonds, respectively. Combined best linear unbiased predictions (BLUPs) and best linear unbiased estimates (BLUEs) for 2023 and 2024 subsets are shown in purple, pink, and orange, respectively. The logarithm of odds (LOD) score is shown on the *y*‐axis, along with the 0.01 and 0.05 LOD thresholds as dashed and solid red lines, respectively. Chromosomes are shown on the *x*‐axis along with marker density. (B) Phenotype by genotype jitter plots of significant markers identified via association mapping in panel A. Homozygous reference (A), heterozygous (H), and homozygous alternative (B) alleles are shown in red, green, and blue, respectively, on the *x*‐axis. The cone openness content is shown on the *y*‐axis. The mean and standard deviation are also shown as a black dot and line, respectively. (C) Favorable allele dosage by phenotype jitter plots of markers with evident additive effect identified in panel B. The number of favorable alleles is on the *x*‐axis, and cone openness and β‐acid content are shown on the *y*‐axis.

To our knowledge, this is the first mapping attempt for cone morphological traits in hop; however, considering the quantitative nature of other hop plant growth traits (McAdam et al., 2014), it is likely that genomic selection for these traits would be preferable over marker assisted selection, like is used for plant sex (Clare et al., 2024). We also identified existing natural allele stacks, or germplasm containing varying numbers of favorable alleles for a given trait within our population. The total number of favorable alleles across individuals was associated with a corresponding advantageous change in the phenotypic trait values (Figures 3, 4, 5, 6, 7). Including the allele effect in the allele stacking analysis to weight more important loci (i.e., categorizing each genotypic class) would likely result in a higher correlation between the phenotype and number of favorable alleles. Therefore, GAB will make it possible to select hop genotypes with natural allele stacks and combine these stacks with other traits of interest such as disease resistance. These results also suggest that the genetic variation present in this population could produce substantial phenotypic improvements for all traits of interest.

Taken together, these results, including high heritability estimates, logical candidate genes, and beneficial natural allele stacks, suggest that the phenotyping methods used in this study were adequate for identifying meaningful associations and that the existing variation in our hop collection is promising for making gains from selection, specifically using GAB approaches. Validation of the NIR method used revealed strong correlations for α‐acids and β‐acids but lower for total oil (Figure 1). Lower prediction accuracy for total oil in hop has been observed in the past (Halsey, 1987). While both NIR and steam distillation methods estimate total oil on an mL/100 g basis, the NIR method appears to be biased toward reliably overestimating quantity. High Spearman rank correlation estimates between the two variables (rho = 0.92; *p* < 0.001) suggest that the method would be adequate for broadly selecting individuals with relatively low and high oil content. This supposition is also supported by the marker results from our study, where some of the candidate genes had logical interpretations, and natural allele stacks resulted in increasing values. Future work should validate molecular markers found using lab‐generated chemistry values and test both methods in genomic prediction models to determine if similar markers are detected. Thus far, evidence would suggest that NIR is reasonable to apply when laboratory work is not feasible due to scale in large mapping experiments. Lab fees for the wet lab chemistry analyses are approximately $85 per sample, whereas we estimate the NIR rental to cost about $3.40 per sample if a user is averaging 70 samples per day and scanning three times per sample as is recommended by the vendor of the machine used in this study. The NIR method also requires minimal equipment overhead and is easy to operate.

Cone size and shape in hop are important traits for selection purposes. Selection intensity at the seedling stage in US public hop breeding can be as high as 1%–5%, partially since appropriate shape,  size, and shatter resistance can exist in low frequencies in certain populations, and these traits are critical for commercial picking processes. In our previous work, we measured openness by dividing cone perimeter by cone length, which appeared to separate out plants with a fluffy cone phenotype versus those with a more conical, tighter morphology (Altendorf et al., 2023). If cone morphology could be predicted, poor performing genotypes with inadequate cones with a likely low picking ability could be culled sooner in the breeding pipeline. Cone shape and size appear to be relatively consistent within a plant and repeatable across plant spacings (Altendorf et al., 2023, 2025). Despite its lower heritability estimate compared with chemistry traits in this study, previous research, as well as the AM results and natural allele stacks presented here, suggest these traits can still be reliably selected in breeding. While the impact of cone shape and size on hop picking, drying, and cone shattering is widely understood in the industry, further work is needed to quantify these relationships to better inform breeding targets.

The large population evaluated here required the use of higher throughput and lower cost phenotyping methods, in addition to a harvest procedure that may not have been perfectly timed for every genotype evaluated. Generally, harvest timing in hop is highly specific to the cultivar and time‐sensitive, as hop chemistry changes as the plants mature (De Keukeleire et al., 2003; Murphy & Probasco, 1996). Considering the scale of the experiment, optimizing harvest timing for each genotype using dry matter evaluations was not feasible. Surprisingly, however, high heritability estimates were still identified despite the subjective‐based harvest methods, suggesting that more precise measurements from plots carefully harvested at peak maturity, and with ASBC wet‐lab measurements, may yield improved associations for breeding purposes. Our previous work also demonstrated that chemistry traits, α‐acids in particular, as well as cone morphology traits, can be reliably selected in first‐year hop seedling evaluations, potentially enabling a tremendous opportunity to realize faster cycle time in hop (Altendorf et al., 2025). Large‐scale seedling evaluations of hop may provide efficient methods for obtaining phenotypic data on cone traits (Altendorf et al., 2025) to further inform GAB approaches in hop. While it is possible to measure α‐acids content in male hop (Nickerson et al., 1988), it is not known in detail how this trait and others transfer into female progeny. Future research should explore the utility of the molecular markers identified here in the selection of superior males as parents in hop crossing schemes (Beatson, 2005).

## 5 CONCLUSIONS

Hop is a critical component of beer production and often marketed as the primary driver of the beer flavor, especially in craft brewing. Therefore, determining the genetic components that contribute to the key hop traits of α‐acid, β‐acid, total oil, cone area, and cone openness can inform breeders of which germplasm to select, possibly reducing the historically long cycle time in hop. Strong and significant correlations between NIR and wet‐lab data, high broad‐sense heritability estimates, and logical candidate genes, all suggest the validity of the methods used and demonstrate the utility of higher‐throughput methodologies for phenotyping in large mapping populations in hop. Furthermore, the quantitative nature of the cone chemistry and morphological traits, and the amenability to allele stacking all indicate that genomic selection may be successfully applied to hop breeding.

## AUTHOR CONTRIBUTIONS

**Shaun J. Clare**: Conceptualization; data curation; formal analysis; investigation; methodology; software; validation; visualization; writing—original draft; writing—review and editing. **Peter Schmuker**: Formal analysis; investigation; methodology; software; writing—original draft; writing—review and editing. **Kayla Altendorf**: Conceptualization; data curation; formal analysis; funding acquisition; investigation; methodology; project administration; resources; supervision; validation; visualization; writing—original draft; writing—review and editing.

## CONFLICT OF INTEREST STATEMENT

The authors declare no conflicts of interest.

## Supporting information



Supporting Information

Supplemental File S1: genotypic data

Supplemental File S2: genetic map

Supplemental File S3: phenotypic data

Supplemental File S4: candidate genes

Supplemental File S5: allele dosage data

Supplemental File S6: R script used for all analyses excluding linkage disequilibrium (LD) decay

Supplemental File S7: R script used for LD decay analyses
