# Supplementary material for: Association mapping for hop cone chemistry and morphology identifies natural beneficial allele stacks
Source: Plant Genome. 2026 Apr 28;19(2):e70238. doi: 10.1002/tpg2.70238 (PMC13125966; doi:10.1002/tpg2.70238)
Supplement: Supplementary file 1 [file TPG2-19-e70238-s006.docx]

**Supplemental Materials**

**Title:** Association mapping for hop cone chemistry and morphology identifies natural beneficial allele stacks

**Authors:** Shaun J. Clare, Peter Schmuker & Kayla Altendorf

**Supplemental Figures:**

**Supplemental Figure S1.** Pearson correlation coefficients between cone morphological and chemistry traits in a diverse collection of hop germplasm over two years in Prosser, WA as measured by near-infrared and image analyses.

**Supplemental Figure S2.** QQ plots for all association mapping models. Colors and symbols correspond to in-text figures.

**Supplemental Figure S3.** Linkage disequilibrium decay (r2) by marker distance (Mb) as calculated from the diversity panel used in the present study for each hop chromosome by facet and genomewide (average).

**Supplemental Tables:**

**Supplemental Table S1:** Variance explained per model term from the linear mixed effect models used to calculate BLUPs over two years of phenotypic data, the ratio of variance explained from the main genotype effect and genotype by year interaction effect, and estimate of broad sense heritability.

| **Trait** | **G** | **GxY** | **Error** | **G vs GxY** | **H^2^** |
| --- | --- | --- | --- | --- | --- |
| α-acid | 13.32 | 0.48 | 5.34 | 27.88 | 0.71 |
| β-acid | 2.50 | 0.16 | 1.23 | 15.96 | 0.67 |
| Total Oil | 0.28 | 0.03 | 0.31 | 8.31 | 0.47 |
| Area | 1.11 | 0.16 | 2.34 | 6.88 | 0.32 |
| Openness | 0.05 | 0.03 | 0.06 | 1.76 | 0.43 |

**Supplemental Table S2.** Mean square deviations (MSD) for each of the principal components (PCs) included, model type across the various traits.

| **PCs** | **Model** | **AA** | **BA** | **TO** | **Openness** | **Area** |
| --- | --- | --- | --- | --- | --- | --- |
| PC0 | BLUP | 0.00006 | 0.00047 | 0.00004 | 0.00044 | 0.00094 |
|  | BLUPboxcox | 0.00033 | 0.00079 | 0.00055 | 0.00019 | 0.00293 |
| PC4 | BLUP | 0.00017 | 0.00009 | 0.00001 | 0.00009 | 0.00075 |
|  | BLUPboxcox | 0.00137 | 0.00082 | 0.00002 | 0 | 0.00017 |
| PC0 | 2023BLUE | 0.00007 | 0.00181 | 0.00006 | 0.00022 | 0.00211 |
|  | 2023BLUEboxcox | 0.0001 | 0.00005 | 0.00004 | 0.00067 | 0.00132 |
| PC4 | 2023BLUE | 0.00002 | 0.00001 | 0.00001 | 0.00195 | 0.00155 |
|  | 2023BLUEboxcox | 0.00117 | 0.00017 | 0.00008 | 0.00028 | 0.00025 |
| PC0 | 2024BLUE | 0.00014 | 0.00018 | 0.00094 | 0.00003 | 0.00008 |
|  | 2024BLUEboxcox | 0.00023 | 0.00003 | 0.00051 | 0.00002 | 0.0017 |
| PC4 | 2024BLUE | 0.00058 | 0.00039 | 0.00004 | 0.00002 | 0.00014 |
|  | 2024BLUEboxcox | 0.00004 | 0.00033 | 0.0006 | 0.00002 | 0.00045 |

**Supplemental Files – will be uploaded to Dryad or GitHub:**

Supplemental File S1 - genotypic data

Supplemental File S2 - genetic map

Supplemental File S3 - phenotypic data

Supplemental File S4 - candidate genes

Supplemental File S5 - allele dosage data

Supplemental File S6 - R script used for all analyses excluding linkage disequilibrium (LD) decay

Supplemental File S7 - R script used for LD decay analyses
